# OCTAL: Optimal Completion of gene trees in polynomial time

**DOI:** 10.1186/s13015-018-0124-5

**Published:** 2018-03-15

**Authors:** Sarah Christensen, Erin K. Molloy, Pranjal Vachaspati, Tandy Warnow

**Affiliations:** Department of Computer Science, University of Illinois at Urbana-Champaign, 201 North Goodwin Avenue, Urbana, IL 61801 USA

**Keywords:** Species trees, Gene trees, Missing data, Multispecies coalescent, Phylogenomics

## Abstract

**Background:**

For a combination of reasons (including data generation protocols, approaches to taxon and gene sampling, and gene birth and loss), estimated gene trees are often incomplete, meaning that they do not contain all of the species of interest. As incomplete gene trees can impact downstream analyses, accurate completion of gene trees is desirable.

**Results:**

We introduce the *Optimal Tree Completion problem*, a general optimization problem that involves completing an unrooted binary tree (i.e., adding missing leaves) so as to minimize its distance from a reference tree on a superset of the leaves. We present *OCTAL*, an algorithm that finds an optimal solution to this problem when the distance between trees is defined using the Robinson–Foulds (RF) distance, and we prove that OCTAL runs in $O(n^2)$ time, where *n* is the total number of species. We report on a simulation study in which gene trees can differ from the species tree due to incomplete lineage sorting, and estimated gene trees are completed using OCTAL with a reference tree based on a species tree estimated from the multi-locus dataset. OCTAL produces completed gene trees that are closer to the true gene trees than an existing heuristic approach in ASTRAL-II, but the accuracy of a completed gene tree computed by OCTAL depends on how topologically similar the reference tree (typically an estimated species tree) is to the true gene tree.

**Conclusions:**

OCTAL is a useful technique for adding missing taxa to incomplete gene trees and provides good accuracy under a wide range of model conditions. However, results show that OCTAL’s accuracy can be reduced when incomplete lineage sorting is high, as the reference tree can be far from the true gene tree. Hence, this study suggests that OCTAL would benefit from using other types of reference trees instead of species trees when there are large topological distances between true gene trees and species trees.

**Electronic supplementary material:**

The online version of this article (10.1186/s13015-018-0124-5) contains supplementary material, which is available to authorized users.

## Background

Species tree estimation from multi-gene datasets is now increasingly common. One challenge is that the evolutionary history for a single locus (called a “gene tree”) may differ from the species phylogeny due to a variety of different biological processes. Some of these processes, such as hybridization [1] and horizontal gene transfer [2], result in non-treelike evolution and so require phylogenetic networks for proper analysis [3–6]. However, other biological processes, such as gene duplication and loss, incomplete lineage sorting (ILS), and gene flow, produce heterogeneity across the genome but are still properly modeled by a single species tree [7, 8]. In the latter case, species tree estimation methods should be robust to heterogeneity across the genome.

Much of the recent focus in the mathematical and statistical phylogenetics literature has been on developing methods for species tree estimation in the presence of incomplete lineage sorting (ILS), which is modelled by the multi-species coalescent (MSC) model [9]. One popular approach for estimating species trees under the MSC model is to estimate trees on individual loci and then combine these gene trees into a species tree. Some of these “summary methods”, such as ASTRAL-II [10] and ASTRID [11], have been shown to scale well to datasets with many taxa (i.e., >1000 species) and provide accurate species tree estimates. (Summary methods share many features in common with supertree methods, but are based on mathematical properties of the MSC model and so can be proven statistically consistent under the MSC model; supertree methods, by contrast, assume conflict between source trees is due to estimation error rather than ILS, and so are generally not statistically consistent under the MSC model.)

A common challenge to species tree estimation methods is that sequence data may not be available for all genes and species of interest, creating conditions with missing data (see discussion in [12–14]). For example, gene trees can be missing species simply because some species do not contain a copy of a particular gene, and in some cases, no common gene will be shared by every species in the set of taxa [15]. Additionally, not all genomes may be fully sequenced and assembled, as this can be operationally difficult and expensive [13, 16].

Although summary methods are statistically consistent under the MSC model [17], the proofs of statistical consistency assume that all gene trees are complete, and so may not apply when the gene trees are missing taxa. Recent extensions to this theory have shown that some species tree estimation methods are statistically consistent under *some* models of missing data (e.g., when “every species is missing from each gene with the same probability $p > 0$”) [18]. However, missing data in biological datasets often violates such models (see discussion in [14]); for example, missing data may be biased towards genes with faster rates of evolution [19]. Furthermore, multi-gene datasets with missing data can be “phylogenetically indecisive”, meaning more than one tree topology can be optimal [20]. Because of concerns that missing data may reduce the accuracy of multi-locus species tree estimation methods, many phylogenomic studies have restricted their analyses to only include genes with most of the species (see discussion in [12, 13, 21]).

We approach the challenge of adding missing species into gene trees by formulating the Optimal Tree Completion problem, where we seek to add the missing species to a gene tree to minimize the distance (defined in some way) to another tree, called a “reference tree”. Since the Robinson–Foulds [22] distance is a common metric for comparing trees (where the Robinson–Foulds distance is the total number of unique bipartitions in the two trees), we specifically address the Robinson–Foulds (RF) Optimal Completion problem, which seeks a completion of the input gene tree with respect to a given reference tree that minimizes the RF distance between the two trees. We then present the Optimal Completion of Incomplete gene Tree Algorithm (OCTAL), a greedy polynomial time algorithm that we prove solves the RF Optimal Completion problem exactly. We also present results from an experimental study on simulated datasets comparing OCTAL to a heuristic for gene tree completion within ASTRAL-II. Finally, we conclude with a discussion of results and future research.

## The Optimal Tree Completion problem

### Terminology

Each edge *e* in an unrooted phylogenetic tree defines a bipartition $\pi _e$ on the leaves of the tree induced by the deletion of *e* (but not its endpoints). Each bipartition is thus a split *A*|*B* of the leaf set into two non-empty disjoint parts, *A* and *B*, that cover the leaf set. The set of bipartitions of a tree *T* is given by *C*(*T*) = {$\pi _e$ : $e \in E(T)$}, where *E*(*T*) is the set of edges for tree *T*. We say that two trees have the same *topology* if they have the same set of bipartitions. When two trees *T* and $T'$ have the same leaf set, then the *Robinson–Foulds* (RF) distance [22] between *T* and $T'$, denoted by RF($T,T'$), is the size of the symmetric difference between *C*(*T*) and $C(T')$. In other words, every bipartition in *T* or $T'$ is either shared between the two trees or is unique to one tree, and the RF distance is the number of bipartitions that appear in exactly one tree. When two trees are binary and on the same leaf set, as is the case in this study, the numbers of bipartitions that are unique to each tree are equal, and each is half the RF distance.

Given tree *T* on leaf set *S*, *T* *restricted* to $R \subseteq S$, denoted by $T|_R$, is the minimal subgraph of *T* that connects all elements of *R*, suppressing nodes of degree two. Note that if *T* contains the bipartition *A*|*B*, $T|_R$ contains the restricted bipartition ($A \cap R)|(B \cap R$). If *T* and $T'$ are two trees with *R* as the intersection of their leaf sets, their *shared edges* are edges whose bipartitions restricted to *R* are in the set $C(T|_R)\cap C(T'|_R)$. Correspondingly, their *unique edges* are edges whose bipartitions restricted to *R* are not in the set $C(T|_R)\cap C(T'|_R)$.

### The RF Optimal Tree Completion problem

The problem we address in this paper is the RF Optimal Tree Completion problem, where the distance between trees is defined by the RF distance, as follows:

- Input: An unrooted binary tree *T* on the full taxon set *S* and an unrooted binary tree *t* on a subset of taxa *R* $\subseteq$ *S*
- Output: An unrooted binary tree $T'$ on the full taxon set *S* with two key properties:
  1. $T'$ is a *S-completion* of *t* (i.e., $T'$ contains all the leaves of *S* and $T'|_R = t$) and
  2. $T'$ minimizes the RF distance to *T* among all *S-completions* of *t*

Note that t and $T|_R$ are both on taxon set *R*, but need not be identical. In fact, the RF distance between these two trees is a lower bound on the RF distance between *T* and $T'$.

## OCTAL: Optimal Completion of incomplete gene Trees ALgorithm

The algorithm begins with input tree *t* and adds leaves one at a time from the set $S \setminus R$ until a tree on the full set of taxa *S* is obtained. To add the first leaf, we choose an arbitrary taxon *x* to add from the set $S \setminus R$. We root the tree $T|_{R \cup \{x\}}$ (i.e., *T* restricted to the leaf set of *t* plus the new leaf being added) at *x*, and then remove *x* and the incident edge; this produces a rooted binary tree we will refer to as $T^{(x)}$ that has leaf set *R*.

We perform a depth-first traversal down $T^{(x)}$ until a shared edge *e* (i.e., an edge where the clade below it appears in tree *t*) is found. Since every edge incident with a leaf in $T^{(x)}$ is a shared edge, every path from the root of $T^{(x)}$ to a leaf has a distinct first edge *e* that is a shared edge. Hence, the other edges on the path from the root to *e* are unique edges.

**Fig. 1:**
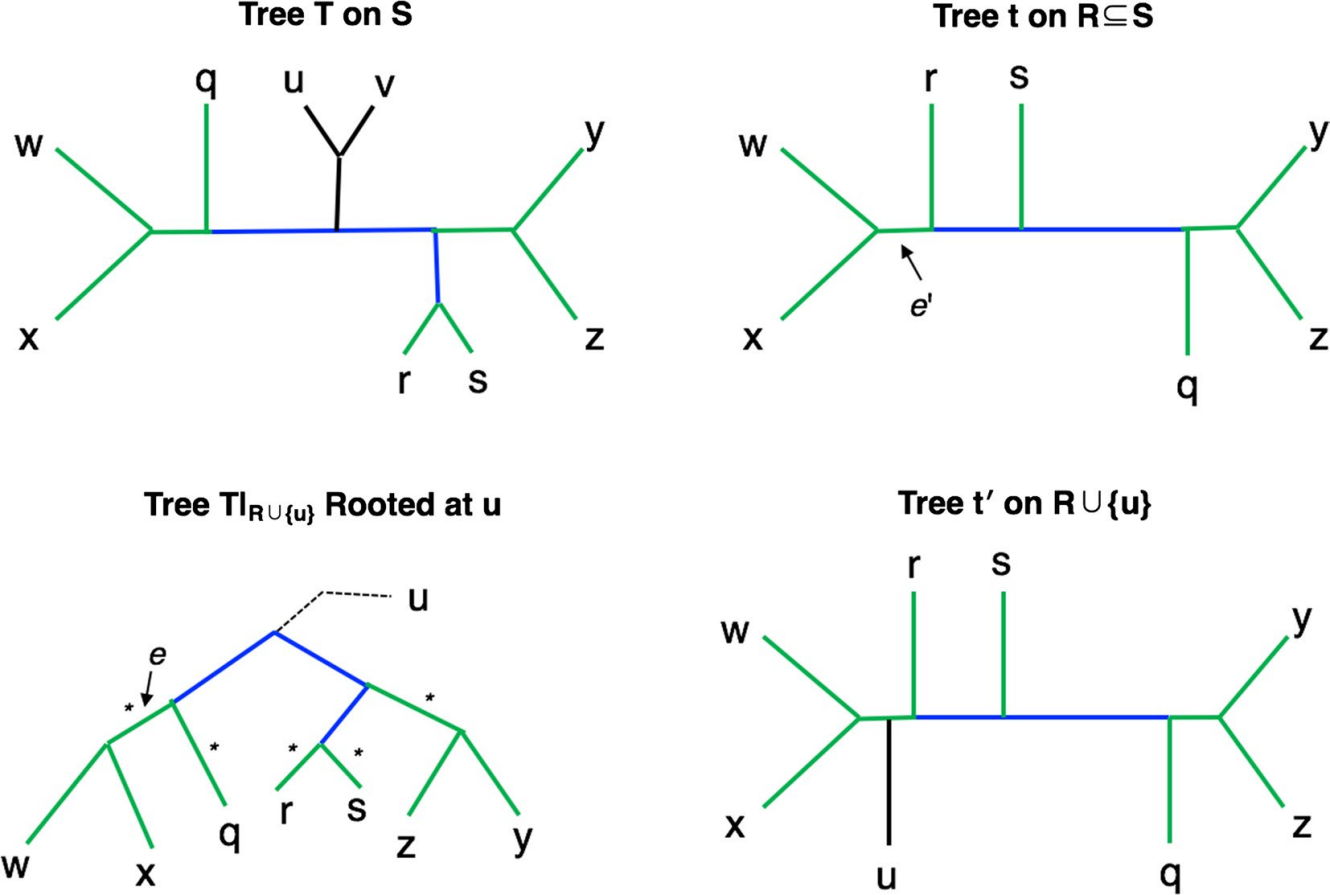
One iteration of the OCTAL algorithm. Trees *T* and *t* with edges in the backbone (defined to be the edges on paths between nodes in the common leaf set) colored green for shared, and blue for unique; all other edges are colored black. After rooting $T|_{R}$ with respect to *u*, the edges in $T|_{R}$ that could be identified by the algorithm for “placement” are indicated with an asterisk (*). Note that any path in $T|_R$ from the root to a leaf will encounter a shared edge, since the edges incident with leaves are always shared. In this scenario, the edge *e* above the least common ancestor of leaves *w* and *x* is selected; this edge defines the same bipartition as edge $e'$ in *t*. Hence, *AddLeaf* will insert leaf *u* into *t* by subdividing edge $e'$, and making *u* adjacent to the newly added node

After we identify the shared edge *e* in $T^{(x)}$, we identify the edge $e'$ in *t* defining the same bipartition, and we add a new node $v(e')$ into *t* so that we subdivide $e'$. We then make *x* adjacent to $v(e')$. Note that since *t* is binary, the modification $t'$ of *t* that is produced by adding *x* is also binary and that $t'|_R = t$. These steps are then repeated until all leaves from $S \setminus R$ are added to *t*. This process is shown in Fig. 1 and given in pseudocode below.

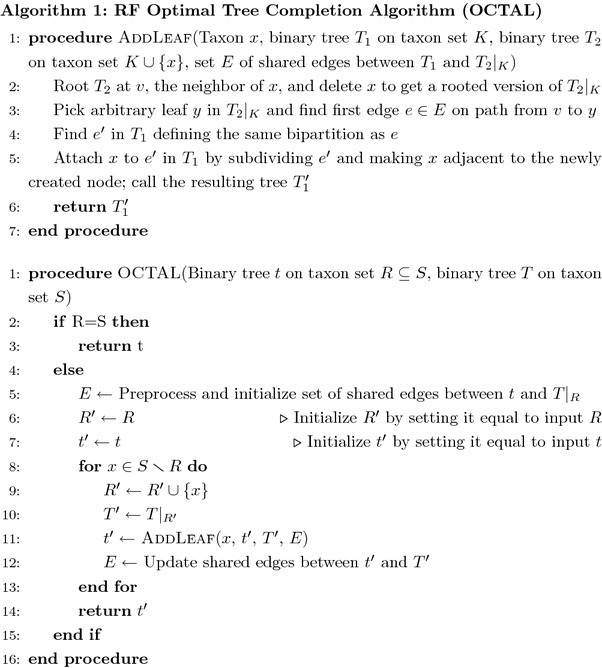



### Proof of correctness

**Fig. 2:**
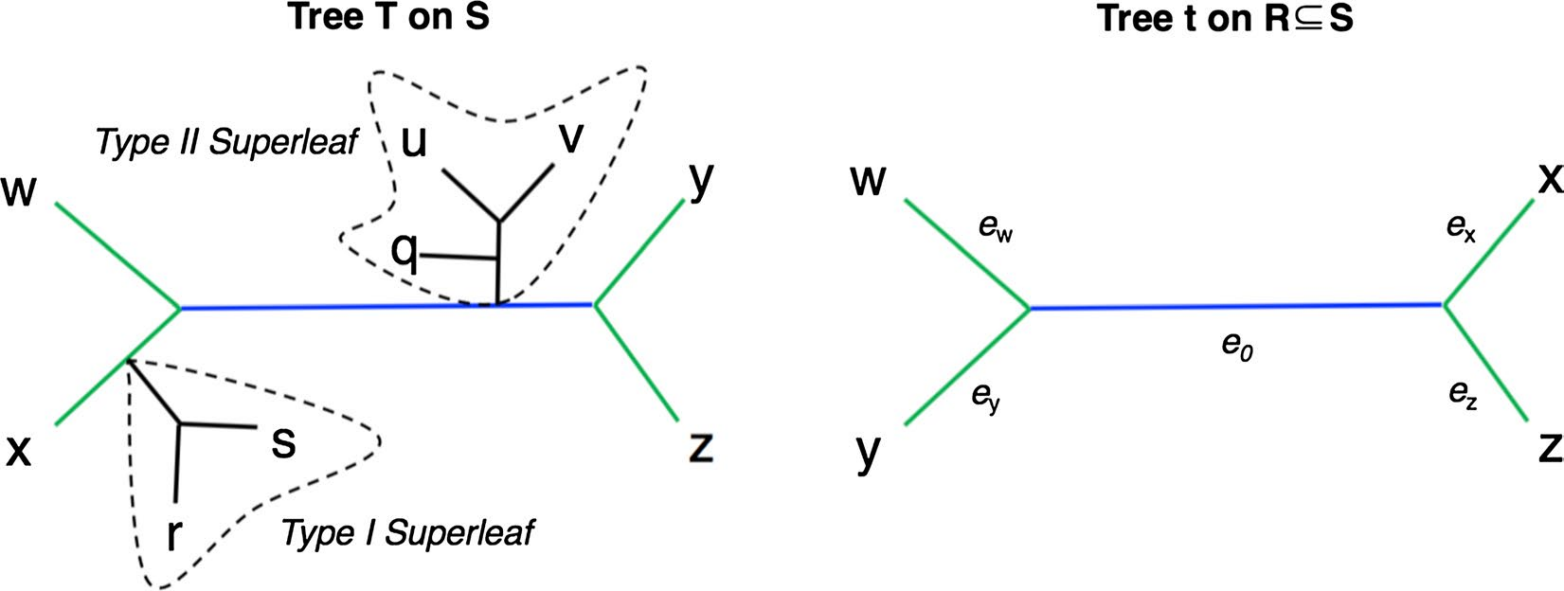
Type I and Type II superleaves. Trees *T* and *t* with edges in the backbone (defined to be the edges on paths between nodes in the common leaf set) colored green for shared, and blue for unique; the other edges are inside superleaves and are colored black. The deletion of the backbone edges in *T* creates two components that are called “superleaves”. One of the two superleaves is a Type I superleaf because it is attached to a shared (green) edge, and the other is a Type II superleaf because it is attached to a unique (blue) edge. The RF distance between *t* and $T|_R$ is equal to 2, the number of blue edges. The Type I superleaf containing leaves *r* and *s* can be added to edge $e_x$ in *t*, the shared edge incident to leaf *x*, without increasing the RF distance; adding it to any other edge in *t* will increase the RF distance by exactly 2. However, adding the Type II superleaf containing leaves *u*, *v*,  and *q* to any single edge in *t* creates exactly one new unique edge in each tree, and therefore increases the RF distance by exactly 2. More generally, for any pair of trees (one a gene tree and the other a reference tree), (1) any Type I superleaf can be added to the gene tree without increasing the RF distance, (2) any addition of a Type II superleaf to the gene tree will always increase the RF distance by *at least* 2, and (3) there is always at least one edge into which a Type II superleaf can be added that increases the RF distance by exactly 2

In what follows, let *T* be an arbitrary binary tree on taxon set *S* and *t* be an arbitrary binary tree on taxon set *R* $\subseteq$ *S*. Let $T'$ denote the tree returned by OCTAL given *T* and *t*. We set $r=RF(T|_R,t)$. As we have noted, OCTAL returns a binary tree $T'$ that is an *S*-completion of *t*. Hence, to prove that OCTAL solves the RF Optimal Tree Completion problem exactly, we only need to establish that $RF(T,T')$ is the smallest possible of all binary trees on leaf set *S* that are *S*-completions of *t*. While the algorithm works by adding a single leaf at a time, we use two types of subtrees, denoted as *superleaves* (see Fig. 2), to aid in the proof of correctness.

#### **Definition 1**

The *backbone* of *T* with respect to *t* is the set of edges in *T* that are on a path between two leaves in *R*.

#### **Definition 2**

A *superleaf* of *T* with respect *t* is a rooted group of leaves from $S \setminus R$ that is attached to an edge in the backbone of *T*. In particular, each superleaf is rooted at the node that is incident to one of the edges in the backbone

#### **Definition 3**

There are exactly two types of superleaves, Type I and Type II:

1. A superleaf is a *Type I superleaf* if the edge *e* in the backbone to which the superleaf is attached is a shared edge in $T|_R$ and *t*. It follows then that a superleaf *X* is a Type I superleaf if and only if there exists a bipartition *A*|*B* in $C(t) \cap C(T|_R)$ where $A|(B \cup X)$ and $(A \cup X)|B$ are both in $C(T|_{R \cup X})$.
2. A superleaf is a *Type II superleaf* if the edge *e* in the backbone to which the superleaf is attached is a unique edge in $T|_R$ and *t*. It follows that a superleaf *X* is a Type II superleaf if and only if for any bipartition *A*|*B* such that $A|(B \cup X)$ and $(A \cup X)|B$ are both in $C(T|_{R \cup X})$, $A|B \not \in C(t)$.

Now we begin our proof by establishing a lower bound on the RF distance to *T* for all binary *S*-completions of *t*.

#### **Lemma 4**

*Let Y be a Type II superleaf for the pair (T, t), and let *$x \in S \setminus R$.* Let *$t^*$ *be the result of adding x into t arbitrarily (i.e., we do not attempt to minimize the resulting RF distance). If *$x \not \in Y$, *then Y is a Type II superleaf for the pair* $(T,t^*)$. *Furthermore, if *$x \in Y$,* then* $RF(T|_{R \cup \{x\}}, t^*) \ge RF(T|_R,t) +2$.

#### *Proof*

It is easy to see that if $x \not \in Y$, then *Y* remains a Type II superleaf after *x* is added to *t*. Now suppose $x \in Y$. We will show that we cannot add *x* into *t* without increasing the RF distance by at least 2. Since *Y* is a Type II superleaf, it is attached to a unique edge in $T|_{R \cup Y}$, and this is the same edge that *x* is attached to in $T|_{R \cup \{x\}}$. So suppose that *x* is added to *t* by subdividing an arbitrary edge $e'$ in *t* with bipartition C|D; note that we do not require that *x* is added to a shared edge in *t*. After adding *x* to *t* we obtain tree $t^*$ whose bipartition set includes $C|(D \cup \{x\})$ and $(C \cup \{x\})|D$. If C|D corresponds to a unique edge relative to *t* and $T|_R$, then both of these bipartitions correspond to unique edges relative to $t^*$ and $T|_{R \cup \{x\}}$. If C|D corresponds to a shared edge, then at most one of the two new bipartitions can correspond to a shared edge, as otherwise we can derive that *Y* is a Type I superleaf. Hence, the number of unique edges in *t* must increase by at least one no matter how we add *x* to *t*, where *x* belongs to a Type II superleaf. Since *t* is binary, the tree that is created by adding *x* is binary, so that $RF(T|_{R \cup \{x\}},t^*) \ge RF(T|_R,t) +2$. $\square$

#### **Lemma 5**

*Let* $T^*$* be an unrooted binary tree that is a S-completion of t. Then *$RF(T^*,T) \ge r+2m$,* where* $r=RF(T|_R,t)$* and m is the number of Type II superleaves for the pair (T, t).*

#### *Proof*

We note that adding a leaf can never reduce the total RF distance. The proof follows from Lemma 4 by induction. $\square$

Now that we have established a lower bound on the best achievable RF distance (i.e., the optimality criterion for the RF Optimal Tree Completion problem), we show OCTAL outputs a tree $T'$ that is guaranteed to achieve this lower bound. We begin by noting that when we add *x* to *t* by subdividing some edge $e'$, creating a new tree $t'$, all the edges other than $e'$ in *t* continue to “exist” in $t'$ although they define new bipartitions. In addition, $e'$ is split into two edges, which can be considered new. Thus, we can consider whether edges that are shared between *t* and *T* *remain* shared after *x* is added to *t*.

#### **Lemma 6**

*Let* $t'$* be the tree created by AddLeaf given input tree t on leaf set R and tree T on leaf set* $R \cup \{x\}$.* If x is added to tree t by subdividing edge* $e'$* (thus creating tree* $t'$),* then all edges in t other than* $e'$* that are shared between t and T remain shared between *$t'$* and T*.

#### *Proof*

Let $T^{(x)}$ be the rooted tree obtained by rooting *T* at *x* and then deleting *x*. Let *e* be the edge in $T^{(x)}$ corresponding to $e'$, and let $\pi _e=A|B$; without loss of generality assume *A* is a clade in $T^{(x)}$. Note that *C*(*T*) contains bipartition $A|(B \cup \{x\})$ (however, *C*(*T*) may not contain $(A \cup \{x\})|B$, unless *e* is incident with the root of $T^{(x)}$). Furthermore, for subclade $A' \subseteq A$, $A'|(R \setminus A') \in$ $C(T|_R)$ and $A'|(R \setminus A' \cup \{x\}) \in$ *C*(*T*). Now suppose $e^*$ in *t* is a shared edge between *t* and $T|_R$ that defines bipartition $C|D \ne A|B$. Since *A*|*B* and *C*|*D* are both bipartitions of *t*, without loss of generality either $C \subset A$ or $A \subset C$. If $C \subset A$, then *C* is a clade in $T^{(x)}$, and so $e^*$ defines bipartition $C|(D \cup \{x\})$ within $t'$. But since $C \subset A$, the previous analysis shows that $C|(D \cup \{x\})$ is also a bipartition of *T*, and so $e^*$ is shared between *T* and $t'$. Alternatively, suppose $A \subset C$. Then within $t'$, $e^*$ defines bipartition $(C \cup \{x\})|D$, which also appears as a bipartition in *T*. Hence, $e^*$ is also shared between *T* and $t'$. Therefore, any edge $e^*$ other than $e'$ that is shared between *t* and *T* remains shared between $t'$ and *T*, for all leaves *x* added by *AddLeaf*. $\square$

#### **Lemma 7**

*OCTAL(T, t) preserves the topology of superleaves in T (i.e. for any superleaf with some subset of leaves* $Q \subseteq S$,* OCTAL(T, t)*$|_Q$* equals* $T|_Q$).

#### *Proof*

We will show this by induction on the number of leaves added. The lemma is trivially true for the base case when just one leaf is added to *t*. Let the inductive hypothesis be that the lemma holds for adding up to *n* leaves to *t* for some arbitrary $n \in \mathbb {N}^+$. Now consider adding $n+1$ leaves, and choose an arbitrary subset of *n* leaves to add to *t*, creating an intermediate tree $t'$ on leaf set *K* using the algorithm OCTAL. Let *x* be the next additional leaf to be added by OCTAL.

If *x* is the first element of a new superleaf to be added, it is trivially true that the topology of its superleaf is preserved, but we need to show that *x* will not break the monophyly of an existing superleaf in $t'$. By the inductive hypothesis, the topology of each superleaf already placed in $t'$ has been preserved. Thus, each superleaf placed in $t'$ has some shared edge in $t'$ and $T|_{K}$ incident to that superleaf. If *x* were placed onto an edge contained in some existing superleaf, that edge would change its status from being shared to being unique, which contradicts Lemma 6.

The last case is where *x* is part of a superleaf for the pair (*T*, *t*) that already has been added in part to *t*. *AddLeaf* roots $T|_{K \cup \{x\}}$ at *x* and removes the edge incident to *x*, creating rooted tree $T^{(x)}$. The edge incident to the root in $T^{(x)}$ must be a shared edge by the inductive hypothesis. Thus, OCTAL will add *x* to this shared edge and preserve the topology of the superleaf. $\square$

#### **Lemma 8**

*OCTAL(T, t) returns binary tree *$T'$* such that *$RF(T,T')=r+2m$,* where m is the number of Type II superleaves for the pair (T, t) and* $r=RF(T|_R,t)$.

#### *Proof*

We will show this by induction on the number of leaves added.

*Base Case* Assume $|S\setminus R|$ = 1. Let *x* be the leaf in S$\setminus R$. *AddLeaf* adds *x* to a shared edge of *t* corresponding to some bipartition A|B, which also exists in $T^{(x)}$.

1. First we consider what happens to the RF distance on the edge *x* is attached to.
  If *x* is a Type I superleaf, the edge incident to the root in $T^{(x)}$ will be a shared edge by the definition of Type I superleaf, so *AddLeaf* adds *x* to the corresponding edge $e'$ in *t*. The two new bipartitions that are created when subdividing $e'$ will both exist in *T* by the definition of Type I superleaf so the RF distance does not change.
  If *x* is a Type II superleaf, either $(A \cup \{x\})|$B or $A|(B\cup \{x\})$ must not exist in *C*(*T*). Since *AddLeaf* adds *x* to a shared edge, exactly one of those new bipartitions must exist in *C*(*T*).
2. Now we consider what happens to the RF distance on the edges *x* is *not* attached to. Lemma 6 shows that *AddLeaf* (and therefore OCTAL) preserves existing shared edges between *t* and $T|_R$, possibly excluding the edge where *x* is added.

Thus, the RF distance will only increase by 2 if *x* is a Type II superleaf, as claimed.

*Inductive step* Let the inductive hypothesis be that the lemma holds for up to *n* leaves for some arbitrary $n \in \mathbb {N}^+$. Assume $|S\setminus R|$ = $n+1$. Now choose an arbitrary subset of leaves $Q\subseteq S \setminus R$, where $|Q|=n$, to add to *t*, creating an intermediate tree $t'$ using the algorithm OCTAL. By the inductive hypothesis, assume $t'$ is a binary tree with the RF distance between $T|_{Q\cup R}$ and $t'$ equal to $r+2m$, where *m* is the number of Type II superleaves in *Q*. *AddLeaf* adds the remaining leaf *x* $\in S\setminus R$ to a shared edge of $t'$ and $T|_{Q\cup R}$.

1. Lemma 6 shows that *AddLeaf* (and therefore OCTAL) preserves existing shared edges between $t'$ and $T|_{Q\cup R}$, possibly excluding the edge where *x* is added.
2. Now we consider what happens to the RF distance on the edge *x* is attached to. There are three cases: (i) *x* is not the first element of a superleaf (ii) *x* is the first element of a Type I superleaf or (iii) *x* is the first element of a Type II superleaf.
  Case (i): If *x* is not the first element of a superleaf to be added to *t*, it directly follows from Lemma 7 that OCTAL will not change the RF distance when adding *x*.
  Case (ii): If *x* is the first element of a Type I superleaf to be added, then *x* is attached to a shared edge in the backbone corresponding to some bipartition *A*|*B* existing in both *C*(*t*) and $C(T|_R)$. Let $e'$ be the edge in *t* s.t. $\pi _{e'}=A|B$. Note there must exist an edge *e* in $T|_{Q\cup R}$ producing *A*|*B* when restricted to just *R*. Hence, the bipartition $\pi _e$ has the form *M*|*N* where $(M \cap R) = A$ and $(N \cap R) = B$. We need to show that $M|N \in C(t')$.
  By Lemma 6, any leaves from *Q* not attached to $e'$ by OCTAL will preserve this shared edge in $t'$.
  Now consider when leaves from *Q* are added to $e'$ by OCTAL. We decompose *M* and *N* into the subsets of leaves existing in either *R* or *Q*: let $M = A \cup W$ and $N = B \cup Z$. OCTAL will not cross a leaf from *W* with a leaf from *Z* along $e'$ because this would require crossing the shared edge dividing these two groups: any leaf $w \in W$ has the property that $(A\cup \{w\}) | B$ is a shared edge and any leaf $z \in Z$ has the property that $A | (B \cup \{z\})$ is a shared edge. Hence, any leaves added from *Q* that subdivide $e'$ will always preserve an edge between leaves contained in *W* and *Z* on $e'$.
  Thus, $M|N \in C(t')$. Moreover, $(M\cup \{x\})| N$ and $M | (N \cup \{x\})$ are bipartitions in *C*(*T*). *AddLeaf* roots *T* at *x* and removes the edge incident to *x*, creating rooted tree $T^{(x)}$. We have shown that the edge incident to the root in $T^{(x)}$ must be a shared edge, so adding *x* does not change the RF distance.
  Case (iii): If *x* is the first element of a Type II superleaf to be added, we have shown in Lemma 4 that the RF distance must increase by at least two. Since *AddLeaf* always attaches *x* to some shared edge $e'$, the RF distance increases by exactly 2 when subdividing $e'$.
  Thus, OCTAL will only increase the RF distance by 2 if *x* is a new Type II superleaf.

$$\square$$

Combining the above results, we establish our main theorem:

#### **Theorem 9**

*Given unrooted binary trees t and T with the leaf set of t a subset of the leaf set of T, OCTAL(T, t) returns an unrooted binary tree* $T'$ *that is a completion of t and that has the smallest possible RF distance to T. Hence, OCTAL finds an optimal solution to the RF Optimal Tree Completion problem. Furthermore, OCTAL runs in* $O(n^2)$ *time, where T has n leaves.*

#### *Proof*

To prove that OCTAL solves the RF Optimal Tree Completion problem optimally, we need to establish that OCTAL returns an *S*-completion of the tree *t*, and that the RF distance between the output tree $T'$ and the reference tree *T* is the minimum among all *S*-completions. Since OCTAL always returns a binary tree and only adds leaves into *t*, by design it produces a completion of *t* and so satisfies the first property. By Lemma 8, the tree $T'$ output by OCTAL has an RF score that matches the lower bound established in Lemma 5. Hence, OCTAL returns a tree with the best possible score among all *S*-completions.

We now show that OCTAL can be implemented to run in $O(n^2)$ time, as follows. The algorithm has two stages: a preprocessing stage that can be completed in $O(n^2)$ time and a second stage that adds all the leaves from $S \setminus R$ into *t* that also takes $O(n^2)$ time.

In the preprocessing stage, we annotate the edges of *T* and *t* as either shared or unique, and we compute a set *A* of pairs of shared edges (one edge from each tree that define the same bipartition on *R*). We pick $r \in R$, and we root both *t* and *T* at *r*. We begin by computing, for each of these rooted trees, the LCA (least common ancestor) matrix for all pairs of nodes (leaves and internal vertices) and the number $n_u$ of leaves below each node *u*; both can be computed easily in $O(n^2)$ time using dynamic programming. (For example, to calculate the LCA matrix, first calculate the set of leaves below each node using dynamic programing, and then calculate the LCA matrix in the second step using the set of leaves below each node.) The annotation of edges in *t* and *T* as shared or unique, and the calculation of the set *A*, can then be computed in $O(n^2)$ time as follows. Given an edge $e \in E(T)$, we note the bipartition defined by *e* as *X*|*Y*, where *X* is the set of leaves below *e* in the rooted version of *T*. We then let *u* denote the LCA of *X* in *t*, which we compute in *O*(*n*) time (using *O*(*n*) LCA queries of pairs of vertices, including internal nodes, each of which uses *O*(1) time since we already have the LCA matrix). Once we identify *u*, we note the edge $e'$ above *u* in *t*. It is easy to see that *e* is a shared edge if and only if *e* and $e'$ induce the same bipartition on *R*, and furthermore this holds if and only if $n_u = |X|$. Hence, we can determine if *e* is a shared edge, and also its paired edge $e'$ in *t*, in *O*(*n*) time. Each edge in *T* is processed in *O*(*n*) time, and hence the preprocessing stage can be completed in $O(n^2)$ time.

After the preprocessing, the second stage inserts the leaves from $S \setminus R$ into *t* using *AddLeaf*, and each time we add a leaf into *t* we have to update the set of edges of *t* (since it grows through the addition of the new leaf) and the set *A*. Recall that when we add $s \in S \setminus R$ into *t*, we begin by rooting *T* at *s*, and then follow a path towards the leaves until we find a first shared edge; this first shared edge may be the edge incident with *s* in *T* or may be some other edge, and we let *e* denote the first shared edge we find. We then use the set *A* to identify the edge $e' \in E(t)$ that is paired with *e*. We subdivide $e'$ and make *s* adjacent to the newly created node. We then update *A*, the set of bipartitions for each tree, and the annotations of the edges of *t* and *T* as shared or unique. By Lemma 6, *AddLeaf* preserves all existing shared edges other than the edge the new leaf *x* is placed on, and these specific edges in *E* can each be updated in *O*(1) time. Furthermore, OCTAL places *x* on a shared edge, bifurcating it to create two new edges. Thus, just two edges need to be checked for being shared, which again can be done in *O*(*n*) as claimed. Thus, adding *s* to *t* and updating all the data structures can be completed in *O*(*n*) time. Since there are at most *n* leaves to add, the second stage can be completed in $O(n^2)$ time. Hence, OCTAL runs in $O(n^2)$ time, since both stages take $O(n^2)$ time. $\square$

## Experimental evaluation

### Overview

We compared OCTAL to the heuristic used in ASTRAL-II [10] for completing incomplete gene trees (see [23] for description), noting however that the ASTRAL-II technique is used to expand the search space explored by ASTRAL-II and does not explicitly attempt to minimize the distance to a reference tree. We used simulated datasets generated for [10] that have heterogeneity between gene trees and species trees due to ILS. To evaluate the accuracy of completed trees, we use three criteria: the normalized RF distance, normalized quartet distance, and the matching distance (see below for details).

We performed three sets of experiments:

- The first set of experiments evaluated the relative and absolute performance of ASTRAL-II and OCTAL for three levels of ILS (moderate, high, and very high) under these three evaluation criteria. The impact of the amount of missing data and gene tree estimation error was also examined.
- The second set of experiments evaluated the impact of the number of genes on the performance of ASTRAL-II and OCTAL. We restricted these experiments to two levels of ILS (moderate and high) and one evaluation criterion (normalized RF distance).
- The third set of experiments evaluated the impact of changing the reference tree on OCTAL. We again restricted these experiments to two levels of ILS (moderate and high) and one evaluation criterion (normalized RF distance).

### Simulated datasets

The datasets used in this simulation study were originally generated for the ASTRAL-II study [10] and then modified for the purpose of this study. The full details of the protocol are described in [10], and briefly summarized here.

#### ASTRAL-II datasets

SimPhy [24] was used to simulate a collection of model species trees and, for each species tree, a collection of gene trees (with branch lengths deviating from a molecular clock) under the multi-species coalescent (MSC) model with varying levels of ILS. We refer to these simulated trees as the true gene trees and true species trees. Under this protocol, the true gene trees contain all the species, and the only cause for discordance between the true gene trees and the true species tree is ILS. For each individual true gene tree, INDELible [25] was used to simulate DNA sequences under the GTR+$\Gamma$ model of evolution without insertions or deletions. The numeric model parameters varied across the gene trees and were determined by drawing from a distribution based on biological datasets. There are 50 replicate datasets per model condition.

#### Our modifications

We restricted the datasets examined in this study, by using only 26 species (one outgroup and 25 out of 200 ingroup taxa) and 200 out of 1000 genes. We examined 20 out of 50 replicate datasets for three model conditions: moderate ILS, high ILS, and very high ILS. We characterize the levels of ILS by the average normalized RF distance, referred to as “AD”, between the true gene trees and the true species tree, calculated using Dendropy v4.2.0 [26]. Across all replicate datasets, the average AD was 10% for the moderate ILS condition, 36% for the high ILS condition, and 75% for the very high ILS condition.

We modified all datasets to ensure that some genes were incomplete, as follows. In each replicate (containing 200 genes), 150 genes were randomly selected to be missing data. In order to determine the number of taxa to be deleted from each gene, we noted the number of taxa in each non-trivial clade in the species tree; this produced a multi-set of numbers that vary between 2 and 20. Then for those genes that were selected to have taxa deleted, we selected a number *n* from the multi-set uniformly at random and selected *n* taxa to be deleted from the gene at random. This produced a set of 150 incomplete gene trees that on average were missing approximately 60% of the species. The estimated gene trees were computed using RAxML v8.2.8 [27] under the GTR+$\Gamma$ model from the resulting alignments (i.e., all the sequences for the complete gene trees, and a subset of the sequences for the incomplete gene trees). This produced a set of 200 estimated gene trees (150 of which were incomplete) for every model condition and replicate dataset.

### Gene tree completion

We used two techniques to complete the incomplete gene trees: the heuristic in ASTRAL-II and OCTAL. For the first set of experiments, ASTRID v1.4 was used to create reference trees for OCTAL. Both OCTAL and ASTRAL-II were run 9000 times (150 incomplete gene trees in each of 20 replicates for three ILS levels).

As the amount of available data could potentially impact the quality of the reference tree used in OCTAL as well as the distance matrix computed by ASTRAL-II, we reduced the number of genes in the second set of experiments. In particular, we restricted the original 200-gene datasets to 25, 50, and 100 genes of which 5, 10, and 25 of these genes were complete, respectively; we also only explored the moderate and high ILS conditions, as these are closer to biological datasets. ASTRID v1.4 was again used to create reference trees for OCTAL, and both OCTAL and ASTRAL-II were run an additional 5400 times.

Finally, in the third set of experiments, we directly evaluated the choice of reference tree on OCTAL by using the true species tree, the ASTRID v1.4 [11] tree, a greedy consensus tree, or a random tree drawn from a uniform distribution. Note that the ASTRID tree was computed on the full set of estimated gene trees (both incomplete and complete), while the greedy consensus tree was computed on the subset of estimated gene trees that were complete. For this final set of experiments, OCTAL was run an additional 18,000 times.

### Evaluation criteria

We report error rates only for gene trees that were *completed* by ASTRAL-II or OCTAL, and we examined three different error metrics: *normalized RF distance, normalized quartet distance,* and *matching distance*. The normalized distances produce values that range from 0 to 1; all three distances return 0 only for those pairs of trees that are topologically identical, and so, low scores are better than large scores. The normalized RF distance between the completed estimated gene trees and the true gene trees was computed using Dendropy v4.2.0. This produces a value between 0 and 1, where 0 indicates that the completed estimated gene tree exactly matches the true gene tree and 1 indicates that the two trees have no common bipartitions. The quartet distance between two trees on the same leaf set considers the quartet topologies induced by restricting each tree to all sets of four leaves (i.e. *n* choose four combinations, where *n* is the number of leaves). The quartet distance is then defined as the number of quartets that induce different topologies in the two trees. The matching distance between two trees on the same leaf set is the weight of a minimum weight perfect matching of their bipartitions, where each edge in the matching is weighted by the number of leaves that must be moved in order to transform one bipartition into its paired bipartition in the other tree [28].

We used one-sided paired Wilcoxon Signed-Rank tests [29] to determine whether using OCTAL (with the ASTRID tree) was significantly better than ASTRAL-II on each replicate dataset. As 20 replicate datasets were tested per model condition, a Bonferroni multiple comparison correction [30] was applied (i.e., *p* values indicating significance must be less than 0.0025).

### Commands

- Maximum likelihood gene trees were estimated using RAxML v8.2.8 (where input is the multiple sequence alignment for a given gene):
raxmlHPC-SSE -m GTRGAMMA -p [seed] -n [name] -s [input]
- The random trees were created as follows. A star tree was created from the complete taxon set (i.e., the taxa in the complete trees). This star tree was then randomly resolved into a binary tree so that “the polytomy will be resolved by sequentially... generating all tree topologies equiprobably” [31]. Specifically, the random tree was generated using Dendropy v4.2.0:
  - from dendropy.simulate import treesim
  - from dendropy.utility import GLOBAL_RNG
  - star_tree = treesim.star_tree(original_taxon_namespace)
  - star_tree.resolve_polytomies(limit=2, update_bipartitions=False, rng=GLOBAL_RNG)
- The greedy consensus trees were computed using Bali-Phy v2.3.8 [32], where the input is the set of 50 complete RAxML trees (i.e., trees on the full taxon set):
trees-consensus –greedy-consensus [input] [output]
- The command for ASTRID v1.4 (input is the full set of 200 RAxML trees):
ASTRID-linux -i [input] -o [output]
- The command for ASTRAL v4.10.2 (input is the full set of 200 RAxML trees):
java -jar astral.4.10.12.jar -i [input] -o [output]
- The normalized RF distances were computed using Dendropy v4.2.0:
  - ne1 = len(tr1.internal_edges(exclude_seed_edge=True))
  - ne2 = len(tr2.internal_edges(exclude_seed_edge=True))
  - [fp, fn] = false_positives_and_negatives(tr1, tr2)
  - rf = float(fp + fn) / (ne1 + ne2)
- The quartet distances were computed using QDist[33]:
  - module load openblas/0.2.8-gcc
  - module load gcc/6.2.0
  - ./qdist tr1 tr2
- The matching distances were computed using code provided by the authors from [28], and now available at [34]:
./matching_distance tr1 tr2 numberofleaves

## Results

### Experiment 1: Performance of OCTAL and ASTRAL-II under three levels of ILS

#### Results under moderate ILS levels

**Fig. 3:**
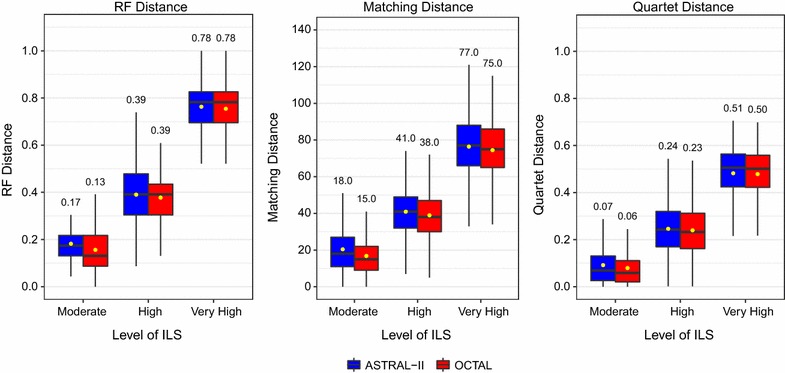
The performance of OCTAL and ASTRAL-II across three levels of ILS evaluated under three tree distance metrics. Each subfigure shows the performance of OCTAL in red (using ASTRID as the reference tree) and ASTRAL-II in blue under one of three distance metrics. Under each distance metric, a lower value indicates the estimated completed tree is closer to the true gene tree. The median distance is reported above each boxplot, and so the outliers are not shown. OCTAL shows the largest improvement over ASTRAL-II under the moderate ILS condition in each case

**Fig. 4:**
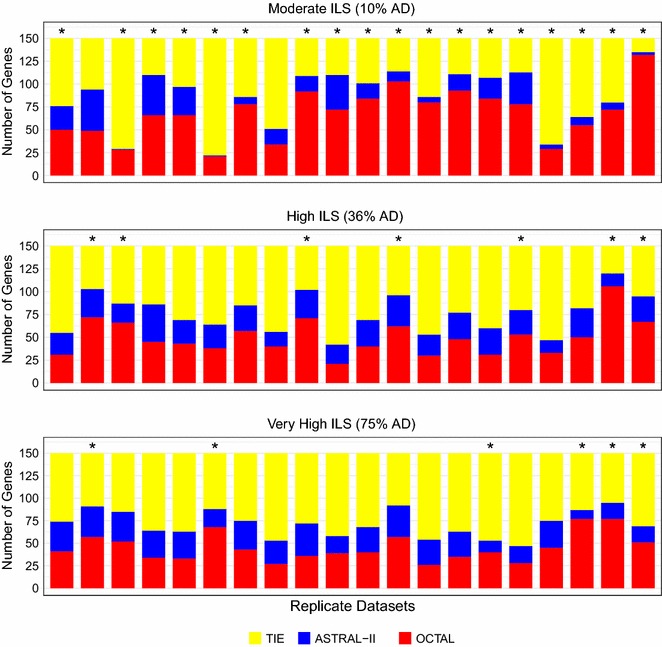
The performance of OCTAL and ASTRAL-II across replicate datasets with the RF distance evaluation criteria. Each subfigure shows the relative performance of OCTAL (using ASTRID as the reference tree) and ASTRAL-II where RF distance was used to compare the estimated completed gene trees to the true gene trees. The number of gene trees for which OCTAL is better than ASTRAL-II is shown in red, the number of gene trees for which ASTRAL-II is better is shown in blue, and the number of genes for which OCTAL and ASTRAL-II are tied is shown in yellow. OCTAL has a statistically significant improvement over ASTRAL-II (as measured by a one-sided Wilcoxon signed-rank test; see main text for details) on replicate datasets with an asterisk (*)

This experiment compared OCTAL (using ASTRID as the reference tree) to ASTRAL-II when given 200 genes (150 incomplete and 50 complete) under the moderate ILS level (AD = 10%). The median RF error rate for ASTRAL-II was 17%, and the median RF error rate for OCTAL was 13% (Fig. 3). Using the RF error rate, OCTAL had better accuracy than ASTRAL-II on 1366 genes, ASTRAL-II had better accuracy on 363 genes, and the methods were tied on the remaining 1271 genes (Table 1). The degree of improvement in RF rate varied, but was as great as 20% on some datasets. The improvement obtained by using OCTAL over ASTRAL-II was statistically significant in 18 out of 20 of the replicates with this evaluation metric (Fig. 4).

**Table 1:** The number of gene trees for which OCTAL provided an improvement over ASTRAL-II, for which ASTRAL-II provided an improvement of OCTAL, and for which there was no difference between the two methods is provided below for three levels of ILS and three evaluation distance criteria

Error metric	OCTAL better	ASTRAL-II better	No difference
Moderate ILS (AD = 10%)			
RF	*1366*	363	1271
Matching	*1666*	522	812
Quartet	*1540*	594	866
High ILS (AD = 35%)			
RF	1004	524	*1472*
Matching	*1501*	920	579
Quartet	*1473*	1092	435
Very high ILS (AD = 75%)			
RF	906	520	*1574*
Matching	*1643*	1143	214
Quartet	*1552*	1371	77

The RF, matching, and quartet distances are used for evaluating the distance between the completed, estimated trees and the true gene trees. Numbers in italic indicate the largest number of genes. OCTAL improves more genes than ASTRAL-II except in the higher ILS conditions with the RF distance criteria, in which case OCTAL and ASTRAL-II are more often equal in their performance

Both the matching distance and quartet distance produced similar trends to the RF distance under the moderate ILS level. The median matching distance was 18 for ASTRAL-II and 15 for OCTAL (Fig. 3) and the improvement obtained by using OCTAL over ASTRAL-II was statistically significant in 19 out of 20 of the replicates (see Additional file 1: Figure S1). The median normalized quartet distance was 7% for ASTRAL-II and 6% for OCTAL (Fig. 3) and the improvement obtained by using OCTAL over ASTRAL-II was statistically significant in 18 out of 20 of the replicates (see Additional file 1: Figure S2).

**Table 2:** The number of gene trees for which OCTAL provided an improvement over ASTRAL-II, for which ASTRAL-II provided an improvement of OCTAL, and for which there was no difference between the two methods is provided below for two levels of ILS and four numbers of genes

Number of genes	OCTAL better	ASTRAL-II better	No difference
Moderate ILS (AD = 10%)			
25 genes	*177*	62	161
50 genes	*420*	116	262
100 genes	*685*	188	627
200 genes	*1366*	363	1271
High ILS (AD = 35%)			
25 genes	*228*	79	93
50 genes	*398*	119	283
100 genes	*624*	265	611
200 genes	1004	524	*1472*

The RF error rate is used for evaluating the distance between the completed, estimated trees and the true gene trees. Numbers in italic indicate the largest number of genes. OCTAL improves more genes than ASTRAL-II except when the level of ILS is high and the number of genes is 200, in which case OCTAL and ASTRAL-II are more often equal in their performance

**Fig. 5:**
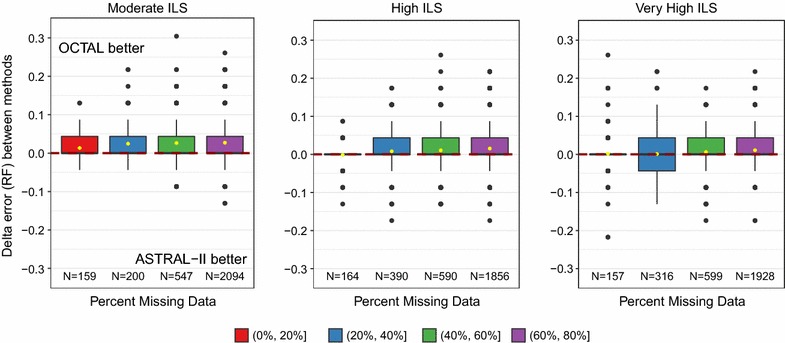
The impact of degree of missing data on relative performance of OCTAL and ASTRAL-II under the RF distance evaluation criteria. The *y*-axis shows the difference in the RF error rate between trees completed using OCTAL (using ASTRID as the reference tree) and ASTRAL-II. Positive values indicate that OCTAL is better than ASTRAL-II, and negative values indicate that ASTRAL-II is better. For many genes, there is no difference in accuracy between OCTAL and ASTRAL-II. However, when there is a difference between the two methods, OCTAL frequently outperforms ASTRAL-II. This finding holds regardless of the degree of missing data. For each level of ILS, boxplots include genes with a specified percent of missing data (e.g., red indicates genes are missing 0–20% of the species). The number *N* of genes in each plot is provided on the *x*-axis

**Fig. 6:**
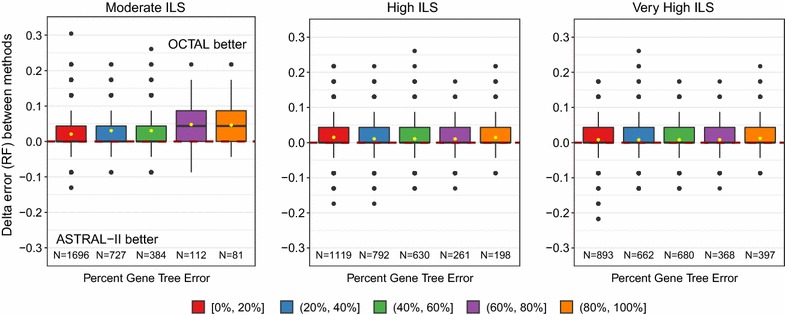
The impact of gene tree estimation error on relative performance of OCTAL and ASTRAL-II under the RF distance evaluation criteria. The *y*-axis shows the difference in the RF error rate between trees completed using OCTAL (using ASTRID as the reference tree) and ASTRAL-II. Positive values indicate that OCTAL is better than ASTRAL-II, and negative values indicate that ASTRAL-II is better. For each level of ILS, boxplots include genes with the specified percent of gene tree estimation error (e.g., red indicates genes have 0–20% RF error). The number *N* of genes in each plot is provided on the *x*-axis

The degrees of missing data and gene tree error did not impact whether OCTAL improved over ASTRAL-II under any of the evaluation metrics. We show our results for missing data with the RF error rate in Fig. 5. Additional results for missing data with the matching distance and quartet distance show the same trend and can be found in Additional file 1: Figures S3 and S4. Under very high levels of gene tree estimation error, there was a greater degree of improvement of OCTAL over ASTRAL-II with the RF error rate (Fig. 6). Additional results for gene tree error with the matching distance and quartet distance show a similar, though less pronounced, trend, and can be found in Additional file 1: Figures S5 and S6.

#### Results under high ILS

This experiment compared OCTAL (using ASTRID as the reference tree) to ASTRAL-II when given 200 genes (150 incomplete and 50 complete) under the high ILS level (AD = 36%). OCTAL and ASTRAL-II achieved similar levels of accuracy under the high ILS condition, with both methods having a median RF error rate of 39% (Fig. 3). OCTAL was more accurate than ASTRAL-II on 1004 genes, ASTRAL-II was more accurate on 524 genes, and the methods were tied on the remaining 1472 genes (Table 1). OCTAL provided a statistically significant advantage over ASTRAL-II in 7 of the 20 replicates, and the differences between the two methods were not statistically significant on the remaining 13 replicates (Fig. 4).

Again, the matching distance and quartet distance produced similar trends to the RF distance. The median matching distance was 41 for ASTRAL-II and 38 for OCTAL (Fig. 3), and the improvement obtained by using OCTAL over ASTRAL-II with respect to the matching distance was statistically significant in 10 out of 20 of the replicates (see Additional file 1: Figure S1). The median normalized quartet distance was 24% for ASTRAL-II and 23% for OCTAL (Fig. 3), and the improvement in quartet distance obtained by using OCTAL over ASTRAL-II was statistically significant in 5 out of 20 of the replicates (see Additional file 1: Figure S2).

Whether OCTAL or ASTRAL-II performed best appeared unrelated to the degree of missing data or gene tree estimation error under all evaluation criteria that we considered. The impact of missing data and the impact of gene tree estimation error on the RF error rate are shown in Figs. 5 and 6, respectively. The results for the matching distance and the quartet distance can be found in Additional file 1: Figures S3–S6.

#### Results under very high ILS

This experiment compared OCTAL (using ASTRID as the reference tree) to ASTRAL-II when given 200 genes (150 incomplete and 50 complete) under the very high ILS level (AD = 75%). Using the RF error rate, OCTAL and ASTRAL-II achieved similar levels of accuracy, with both methods having a substantially increased median RF error rate of 78% (Fig. 3). OCTAL was more accurate than ASTRAL-II on 906 genes, ASTRAL-II was more accurate on 520 genes, and the methods were tied on the remaining 1574 genes. OCTAL provided a statistically significant advantage over ASTRAL-II with the RF error rate in only 6 of the 20 replicates (Fig. 4).

In this case, the median matching distance was 77 for ASTRAL-II and 75 for OCTAL (Fig. 3), and the improvement obtained by using OCTAL over ASTRAL-II was statistically significant in 8 out of 20 of the replicates using the matching distance (see Additional file 1: Figure S1). The median normalized quartet distance was 51% for ASTRAL-II and 50% for OCTAL (Fig. 3) and the improvement in quartet distance obtained by using OCTAL over ASTRAL-II was statistically significant in 2 out of 20 of the replicates (see Additional file 1: Figure S2).

As we observed for the other ILS conditions, whether OCTAL or ASTRAL-II performed best appears unrelated to the degree of missing data or gene tree estimation error with respect to all evaluation criteria we considered. For the impact on RF error rate, Fig. 5 shows results for missing data and Fig. 6 shows results for gene tree error. The remaining results for the matching distance and the quartet distance can be found in Additional file 1: Figures S3–S6.

### Experiment 2: Impact of the number of genes on performance of ASTRAL-II and OCTAL

**Fig. 7:**
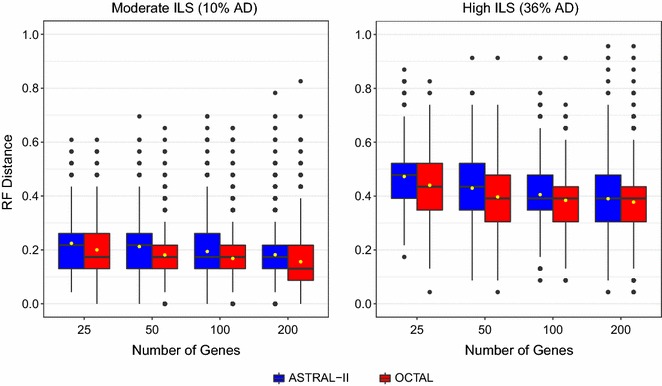
The performance of OCTAL and ASTRAL-II for varying numbers of genes under the RF distance evaluation criteria. The *x*-axis shows the number of genes varying from 25 to 200. The *y*-axis shows the RF error rate between the true gene trees and the gene trees completed using OCTAL with the ASTRID reference tree (red) or ASTRAL-II (blue). The number of data points per boxplot varies with the number of genes. For example, the 25-genes model condition has 400 data points per boxplot (20 incomplete genes across 20 replicates), whereas the 200-gene model condition has 3000 data points per boxplot (150 incomplete genes across 20 replicates)

As the number of genes determines the amount of data to be used in constructing a reference tree (required by OCTAL) and a distance matrix (required by ASTRAL-II), we varied the number of genes to see if this would impact the performance of OCTAL (using ASTRID as the reference tree) or ASTRAL-II under the moderate and high ILS conditions. Specifically, we examined subsets of the original 200-gene datasets with 25, 50, and 100 genes, of which 5, 10, and 25 were complete, respectively. As seen in Fig. 7, under moderate ILS (AD = 10%), ASTRAL-II had a median RF error rate of 22% (for 25 and 50 genes) and 17% (for 100 and 200 genes), whereas OCTAL had a median RF error rate of 17% (for 25, 50, and 100 genes) and 13% (for 200 genes). Hence, OCTAL was generally more accurate (as measured by the RF error rate) than ASTRAL-II under the moderate ILS condition. The relative improvement of OCTAL over ASTRAL-II per gene tree was $7 \pm 4\%$ (mean ± standard deviation) (i.e., 1–2 bipartitions) for all numbers of genes; however, the number of cases for which OCTAL improved over ASTRAL-II varied with the number of genes (see Table 2).

**Fig. 8:**
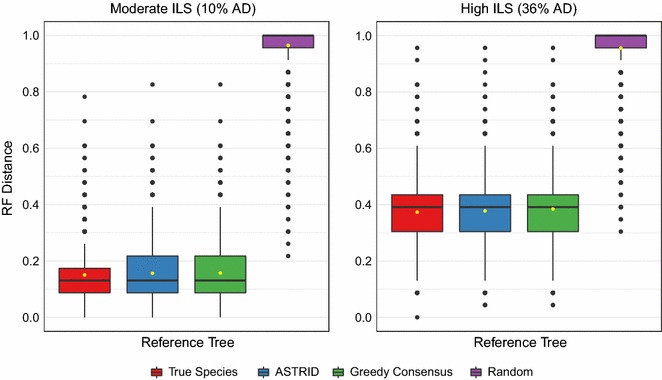
Impact of reference tree on OCTAL with the RF distance evaluation metric. The *x*-axis shows the reference tree used by OCTAL. The *y*-axis shows the RF error rate between the true gene trees and the gene trees computing using OCTAL (varying the reference tree). Only the 200-gene model condition is shown, so each boxplot has 3000 data points (150 incomplete genes across 20 replicates)

Results under high ILS (AD = 36%) show somewhat different trends. ASTRAL-II had a median RF error rate of 48% for 25 genes, 44% for 50 genes, and 39% for 100 and 200 genes. OCTAL had lower median error rates at 25 (44 and 39%, respectively) but matched the median error rates of ASTRAL-II at 100 and 200 genes. However, OCTAL and ASTRAL-II have clearly different distributions for 200 genes (Figs. 3 and 7), so that even though the medians are the same OCTAL seems to provide a slight advantage over ASTRAL-II. Thus, on the high ILS datasets, OCTAL provided an improvement over ASTRAL-II, and the relative improvement per gene tree was similar to performance under the moderate ILS level (7–8% on average); however, there were fewer genes for which OCTAL improved over ASTRAL-II (see Table 2).

### Experiment 3: Impact of the reference tree on the accuracy of OCTAL

Our final experiment examined the impact of reference tree on OCTAL on the 200-gene datasets with moderate and high levels of ILS, using the RF error rate as the evaluation criterion. We considered four reference trees: (1) the true species tree, (2) the ASTRID species tree computed on the all gene trees (50 complete and 150 incomplete), (3) the greedy consensus tree computed on the 50 complete gene trees, and (4) a random tree on the same set of species. The greedy consensus tree, also known as the extended majority consensus tree, is obtained by ordering the bipartitions from the input set of trees according to their frequency, and then adding them one-by one-to a growing set of bipartitions if they are compatible with the set.

The ASTRID and greedy consensus trees had low species tree RF error (at most 9% RF) under the moderate ILS condition and somewhat higher species tree error (at most 22% RF) when the level of ILS was high. We found that there was little difference (less than 1% in median gene tree RF error) between using ASTRID, a greedy consensus of the complete gene trees, and even the true species tree, as the reference tree (Fig. 8). However, using a random tree as the reference tree produced extremely high RF error rates for the completed trees, which is as expected as the random species tree had extremely high error: between 96 and 100% RF for each replicate.

## Discussion

These results show that OCTAL was generally at least as accurate as ASTRAL-II at completing gene trees, and can be more accurate; this trend does not appear to be sensitive to the distance measure used to evaluate the accuracy of the completed gene trees. Within the scope of our study, the degree and frequency of improvement depended on the level of ILS, but not so much on the number of genes or on the reference tree, as long as the reference tree was estimated from the gene trees. Furthermore, using several techniques to produce the reference tree from the gene trees, including even a greedy consensus tree, produced reference trees that were as good as the true species tree in terms of the impact on the accuracy of the completed gene tree. However, a random tree produced very poor results. We also noted that OCTAL provided a clear advantage over ASTRAL-II under low to moderate ILS, but the improvement was smaller and less frequent under the high to very high ILS conditions. We offer the following as a hypothesis for the reason for these trends. Under low to moderate ILS, the true species tree is close to the true gene tree, and the estimated species trees (computed using ASTRID or the greedy consensus) are reasonably close to the true species tree; by the triangle inequality, the estimated species tree is close to the true gene trees. Therefore, when ILS is at most moderate, completing the estimated gene trees using the estimated species tree as a reference can be beneficial. However, under higher ILS, the true species tree is farther from the true gene trees, which makes the true species tree (or an estimate of that tree) less valuable as a reference tree. Despite this, we also saw that using estimated species trees as reference trees produced comparably accurate completions as using the true species tree as a reference, and that this held for both moderate and high ILS levels. Hence, OCTAL was robust to moderate levels of error in the estimated species tree. However, OCTAL is not completely agnostic to the choice of reference tree, since the random reference tree (which has close to 100% RF error) resulted in very poor performance.

## Conclusions

OCTAL is a greedy polynomial time algorithm that adds species into an estimated gene tree so as to provably minimize the RF distance to a given reference tree. In our study, OCTAL frequently produced more accurate completed gene trees than ASTRAL-II under ILS conditions ranging from moderate to very high; however, the improvement under high ILS conditions was much lower and less frequent than under moderate ILS conditions.

There are many directions for future work. First, we compared OCTAL to ASTRAL-II, but ASTRAL-III [35] has recently been developed, and the comparison should be made to this new version of ASTRAL. OCTAL could also be compared to gene tree completion methods that are designed to handle gene tree heterogeneity resulting from gene duplication and loss [36], and these comparisons could be made on datasets that have evolved under multiple causes of gene tree discord (e.g., gene duplication and loss, horizontal gene transfer, and incomplete lineage sorting).

The current approach only adds missing species to the estimated gene tree, and so implicitly assumes that the gene tree is accurate; since estimated gene trees have some error, another approach would allow the low support branches in gene trees to be collapsed and then seek a complete gene tree that refines the collapsed gene tree that is close to the reference tree. This is similar to approaches used in [37–39], each of which aims to improve gene trees that use reference species trees, but are primarily (or exclusively) based on gene duplication and loss (GDL) distances. The optimal completion problem or the accuracy of the completed gene trees could also be based on other distances between trees besides the RF distance, including weighted versions [40] of the RF distance (where the weights reflect branch lengths or bootstrap support values), quartet tree distances, geodesic distances [41], or the matching distance. It is likely that some of these problems will be NP-hard, but approximation algorithms or heuristics may be useful in practice.

We did not evaluate the impact of using OCTAL on downstream analyses. Since missing data (i.e., incomplete gene trees) are known to impact species tree estimation methods using summary methods [21], this would be a natural next analysis. As an example, if the input includes some incomplete gene trees, a species tree could be estimated from the full set of gene trees and then OCTAL could use that estimated species tree as a reference tree to complete the gene trees. Then, the species tree could be re-estimated (using a good summary method) on the new set of gene trees, all of which are complete. This two-step process (completing gene trees using an estimated species tree, then re-estimating the species tree) could then iterate. It would be interesting to determine whether this improves the species tree, and if so under what conditions. It would also be helpful to evaluate the impact of completing incomplete gene trees when the genes are missing due to true biological loss rather than data collection issues, and hence also to see if OCTAL provides any helpful insight into gene evolution (such as better estimating the duplication/loss/transfer parameters).

Finally, there can be multiple optima to the RF Optimal Tree Completion problem for any given pair of trees, and exploring that set of optimal trees could be important. An interesting theoretical question is whether the set of optimal solutions admits a compact representation, even when it is large. From a practical perspective, the set of optimal completions could be used to provide support values for the locations of the missing taxa, and these support values could then be used in downstream analyses.

## Additional file

**Additional file 1.** Additional materials containing Figures S1–S6.

## Abbreviations

AD: average distance between the true species tree and the true gene trees, using the normalized RF metric
GDL: gene duplication and loss
ILS: incomplete lineage sorting
MSC: multi-species coalescent
RF: Robinson–Foulds
