# Supplementary material for: OCTAL: Optimal Completion of gene trees in polynomial time
Source: Algorithms Mol Biol. 2018 Mar 15;13:6. doi: 10.1186/s13015-018-0124-5 (PMC5853121; doi:10.1186/s13015-018-0124-5)
Supplement: Supplementary file 1 — Additional file 1. Additional materials containing Figures S1–S6. [file 13015_2018_124_MOESM1_ESM.pdf]

Additional Materials

OCTAL: Optimal Completion of Gene Trees in Polynomial Time

Sarah Christensen, Erin K. Molloy, Pranjal Vachaspati, and Tandy Warnow

List of Figures

S1 The performance of OCTAL and ASTRAL-II across replicate datasets with the matching distance evaluation criterion 2

S2 The performance of OCTAL and ASTRAL-II across replicate datasets with the quartet distance evaluation criterion 3

S3 The impact of degree of missing data on the relative performance of OCTAL and ASTRAL-II with the matching distance evaluation criterion 4

S4 The impact of degree of missing data on the relative performance of OCTAL and ASTRAL-II with the quartet distance evaluation criterion 5

S5 The impact of gene tree estimation error on the relative performance of OCTAL and ASTRAL-II with the matching distance evaluation criterion 6

S6 The impact of gene tree estimation error on the relative performance of OCTAL and ASTRAL-II with the quartet distance evaluation criterion 7

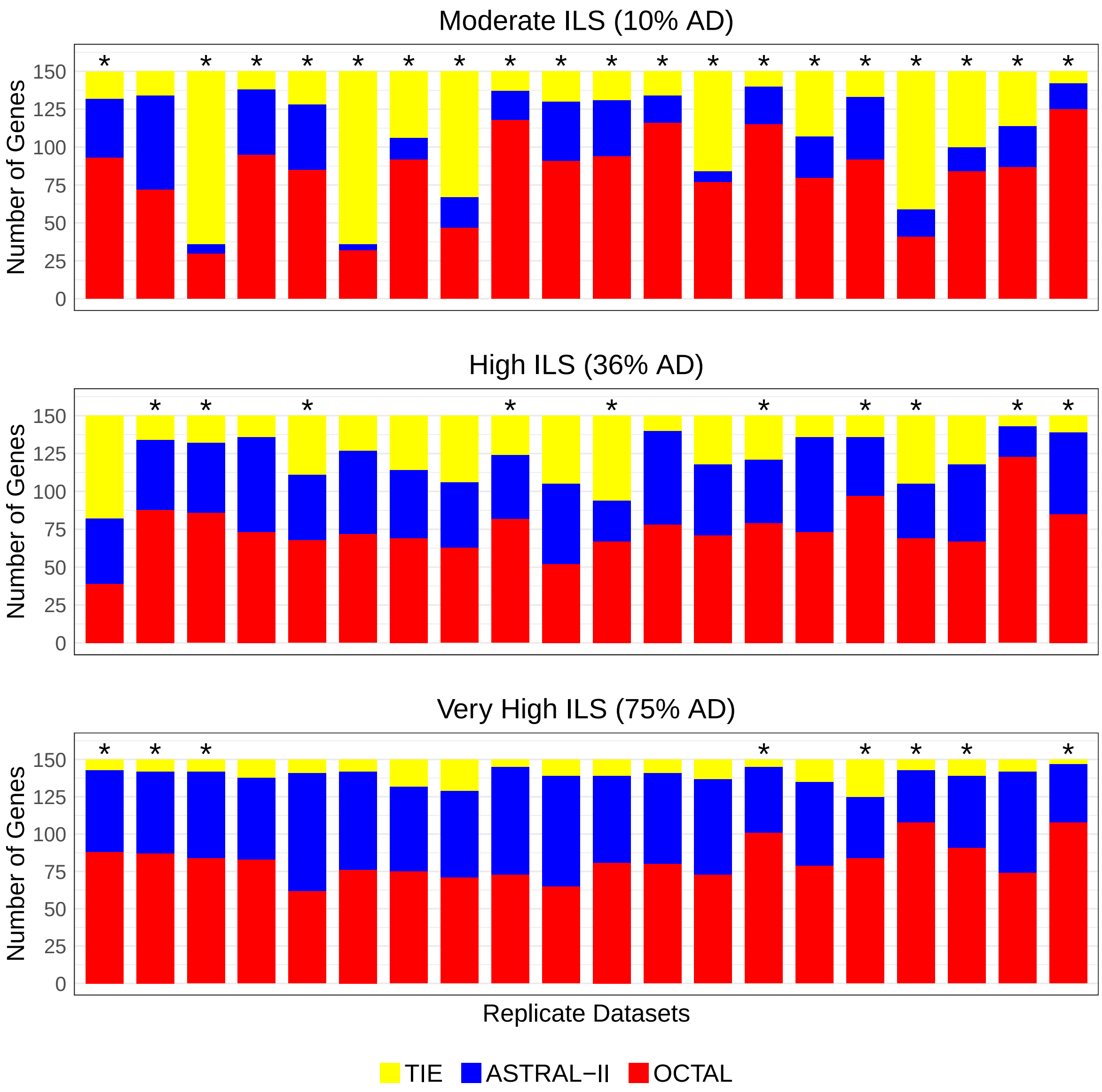

Figure S1: **The performance of OCTAL and ASTRAL-II across replicate datasets with the matching distance evaluation criterion.** Each subfigure shows the relative performance of OCTAL (using ASTRID as the reference tree) and ASTRAL-II where the matching distance was used to compare the estimated, completed gene trees to the true gene trees. The number of gene trees for which OCTAL is better than ASTRAL-II is shown in red, the number of gene trees for which ASTRAL-II is better is shown in blue, and ties are indicated by yellow. OCTAL has a statistically significant improvement over ASTRAL-II on replicates indicated with an asterisk (*).

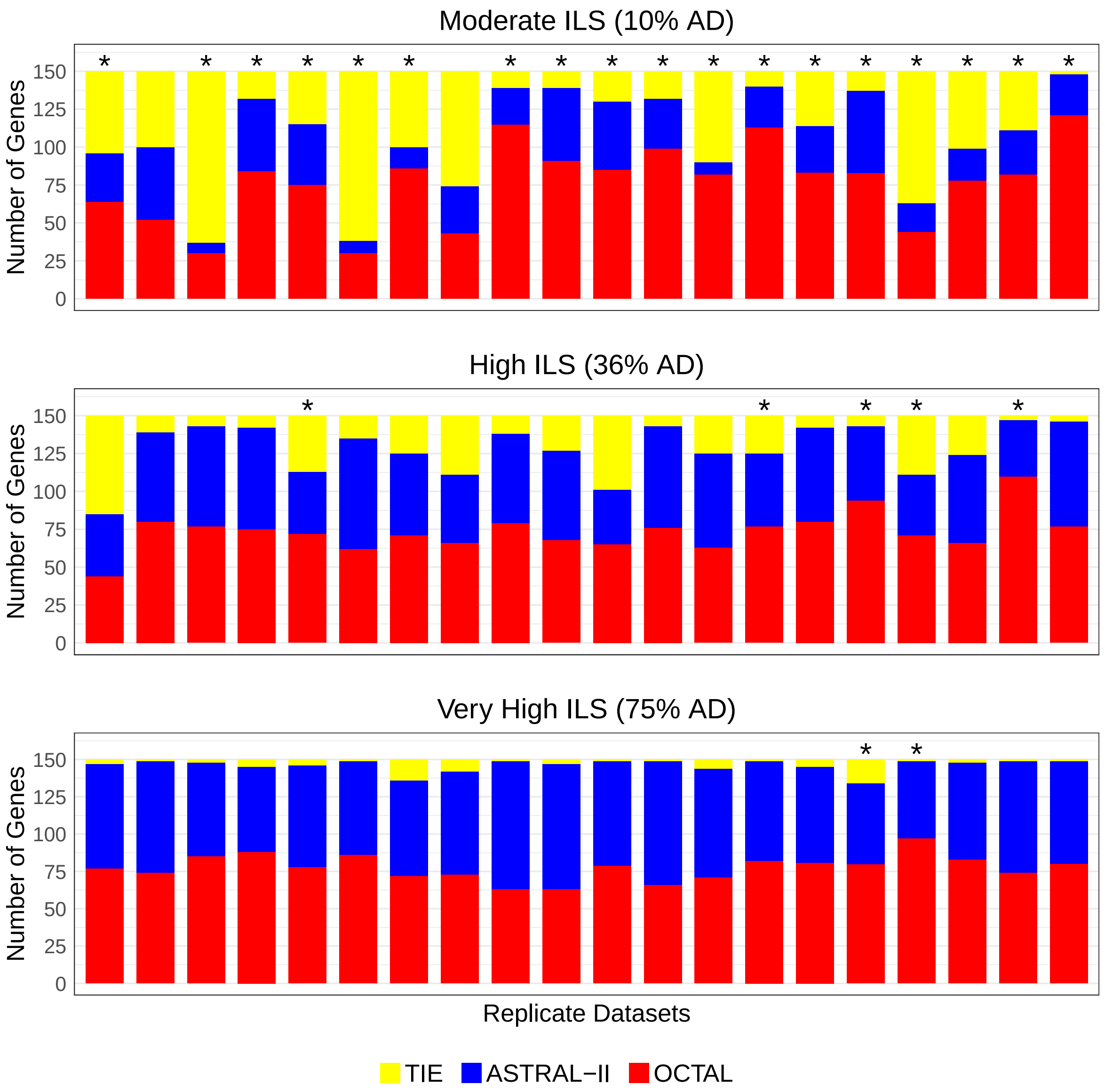

Figure S2: **The performance of OCTAL and ASTRAL-II across replicate datasets with the quartet distance evaluation criterion.** Each subfigure shows the relative performance of OCTAL (using ASTRID as the reference tree) and ASTRAL-II where the quartet distance was used to compare the estimated, completed gene trees to the true gene trees. The number of gene trees for which OCTAL is better than ASTRAL-II is shown in red, the number of gene trees for which ASTRAL-II is better is shown in blue, and ties are indicated by yellow. OCTAL has a statistically significant improvement over ASTRAL-II on replicates indicated with an asterisk (*).

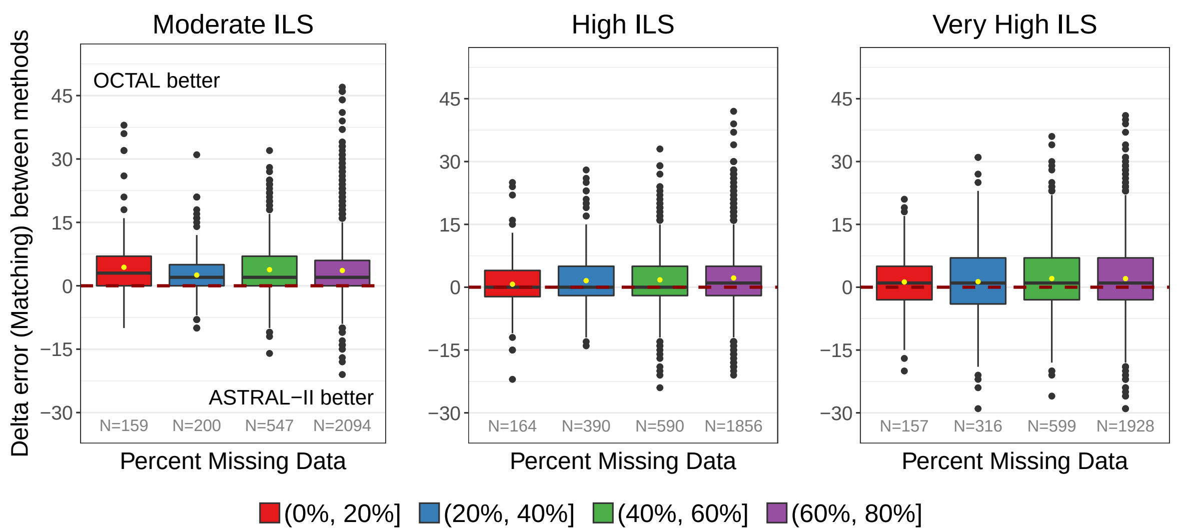

Figure S3: **The impact of degree of missing data on the relative performance of OCTAL and ASTRAL-II with the matching distance evaluation criterion.** The y-axis shows the difference in the matching distance between trees completed using OCTAL (using ASTRID as the reference tree) and ASTRAL-II. Positive values indicate that OCTAL is better than ASTRAL-II, and negative values indicate that ASTRAL-II is better. Each boxplot includes genes with the specified percent of missing data (e.g., red indicates genes are missing 0-20% of the species) for a given level of ILS. The number N of genes in each plot is also provided on the x-axis.

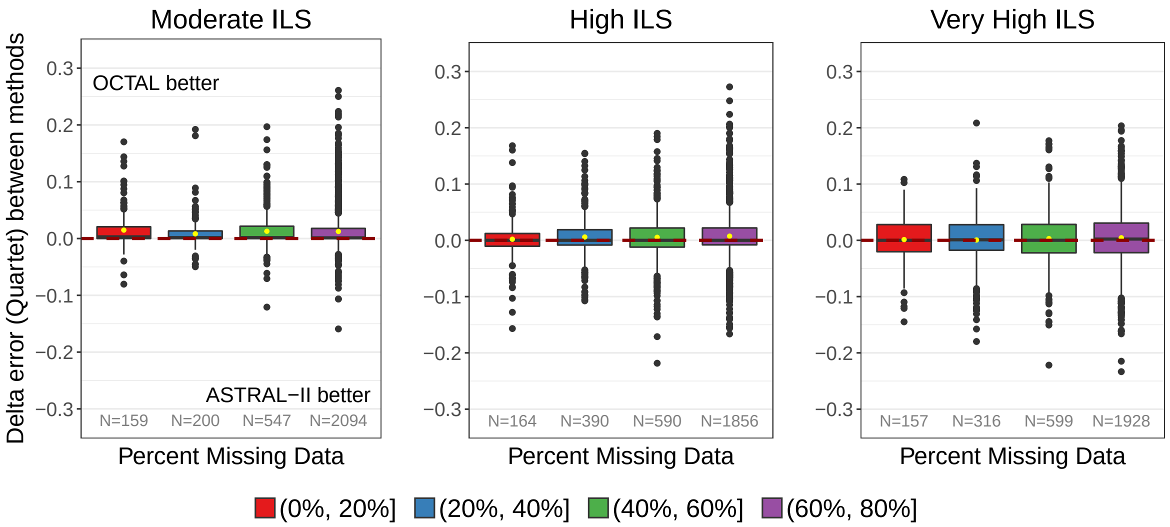

Figure S4: **The impact of degree of missing data on the relative performance of OCTAL and ASTRAL-II with the quartet distance evaluation criterion.** The y-axis shows the difference in the matching distance between trees completed using OCTAL (using ASTRID as the reference tree) and ASTRAL-II. Positive values indicate that OCTAL is better than ASTRAL-II, and negative values indicate that ASTRAL-II is better. Each boxplot includes genes with the specified percent of missing data (e.g., red indicates genes are missing 0-20% of the species) for a given level of ILS. The number N of genes in each plot is also provided on the x-axis.

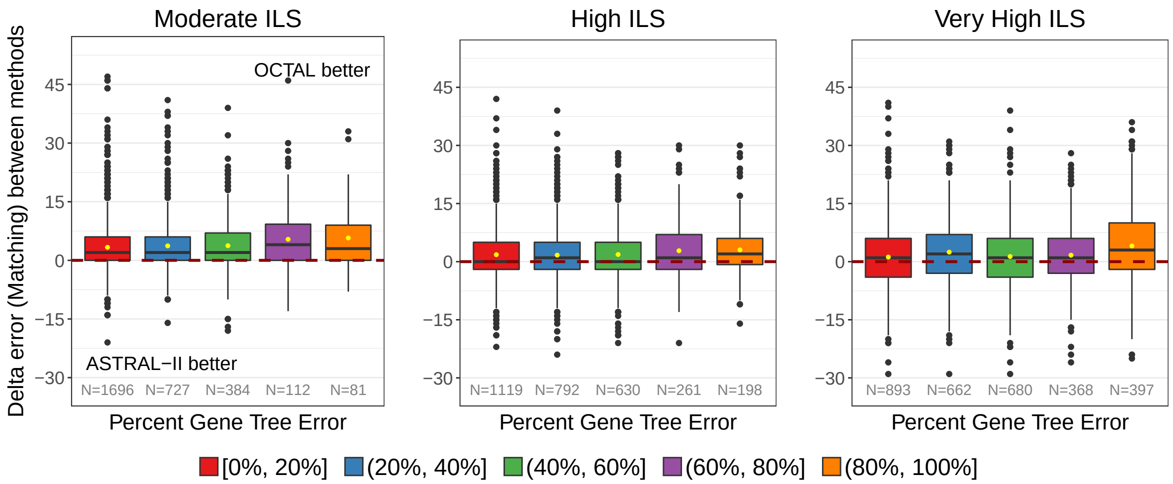

Figure S5: **The impact of gene tree estimation error on the relative performance of OCTAL and ASTRAL-II with the matching distance evaluation criterion.** The y-axis shows the difference in the matching distance between trees completed using OCTAL (using ASTRID as the reference tree) and ASTRAL-II. Positive values indicate that OCTAL is better than ASTRAL-II, and negative values indicate that ASTRAL-II is better. Each boxplot includes genes with the specified percent of gene tree estimation error (e.g., red indicates genes have 0-20% RF error) for a given level of ILS. The number N of genes in each plot is also provided on the x-axis.

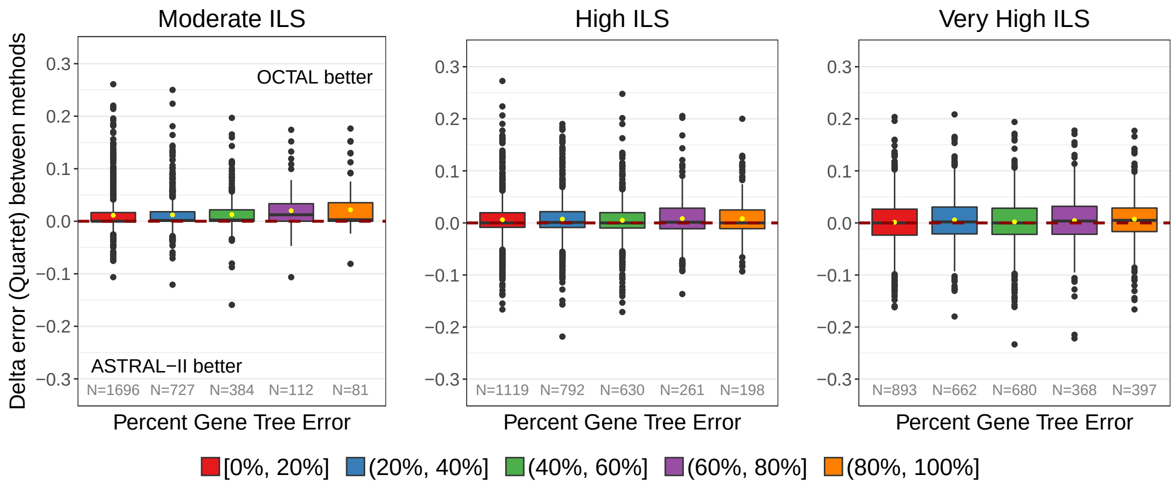

Figure S6: **The impact of gene tree estimation error on the relative performance of OCTAL and ASTRAL-II with the quartet distance evaluation criterion.** The y-axis shows the difference in the quartet distance between trees completed using OCTAL (using ASTRID as the reference tree) and ASTRAL-II. Positive values indicate that OCTAL is better than ASTRAL-II, and negative values indicate that ASTRAL-II is better. Each boxplot includes genes with the specified percent of gene tree estimation error (e.g., red indicates genes have 0-20% RF error) for a given level of ILS. The number N of genes in each plot is also provided on the x-axis.
